# Differential miRNA Expression in Human Macrophage-Like Cells Infected with *Histoplasma capsulatum* Yeasts Cultured in Planktonic and Biofilm Forms

**DOI:** 10.3390/jof7010060

**Published:** 2021-01-18

**Authors:** Nayla de Souza Pitangui, Junya de Lacorte Singulani, Janaina de Cássia Orlandi Sardi, Paula Carolina de Souza, Gabriela Rodríguez-Arellanes, Blanca Estela García-Pérez, Francisco Javier Enguita, Fernando R. Pavan, Maria Lucia Taylor, Maria José Soares Mendes-Giannini, Ana Marisa Fusco-Almeida

**Affiliations:** 1 School of Pharmaceutical Sciences, São Paulo State University (UNESP), Araraquara, São Paulo 14800-903, Brazil; napitangui@hotmail.com (N.d.S.P.); junyadelacorte@yahoo.com.br (J.d.L.S.); paula_farma85@yahoo.com.br (P.C.d.S.); fernandopavan@fcfar.unesp.br (F.R.P.); gianninimj@gmail.com (M.J.S.M.-G.); 2 Department of Cellular and Molecular Biology, Ribeirão Preto School of Medicine, University of São Paulo, Ribeirão Preto, São Paulo 14049-900, Brazil; 3 School of Pharmaceutical Sciences, Food and Nutrition, Federal University of Mato Grosso do Sul, Campo Grande, Mato Grosso do Sul 79070-900, Brazil; janasardi@gmail.com; 4 Unidad de Micología, Departamento de Microbiología y Parasitología, Facultad de Medicina, UNAM-Universidad Nacional Autónoma de México, Mexico City 04510, Mexico; batgaby@unam.mx (G.R.-A.); emello@unam.mx (M.L.T.); 5 Departamento de Microbiología, Escuela Nacional de Ciencias Biológicas, IPN–Instituto Politécnico Nacional, Mexico City 11340, Mexico; abrilestela@hotmail.com; 6 Instituto de Medicina Molecular, Faculdade de Medicina, Universidade de Lisboa, Lisboa 1649-028, Portugal; fenguita@medicina.ulisboa.pt

**Keywords:** *Histoplasma capsulatum*, biofilms, macrophages, microRNAs, fungal-host interactions

## Abstract

*Histoplasma capsulatum* affects healthy and immunocompromised individuals, sometimes causing a severe disease. This fungus has two morphotypes, the mycelial (infective) and the yeast (parasitic) phases. MicroRNAs (miRNAs) are small RNAs involved in the regulation of several cellular processes, and their differential expression has been associated with many disease states. To investigate miRNA expression in host cells during *H. capsulatum* infection, we studied the changes in the miRNA profiles of differentiated human macrophages infected with yeasts from two fungal strains with different virulence, EH-315 (high virulence) and 60I (low virulence) grown in planktonic cultures, and EH-315 grown in biofilm form. MiRNA profiles were evaluated by means of reverse transcription-quantitative polymerase chain reaction using a commercial human miRNome panel. The target genes of the differentially expressed miRNAs and their corresponding signaling pathways were predicted using bioinformatics analyses. Here, we confirmed biofilm structures were present in the EH-315 culture whose conditions facilitated producing insoluble exopolysaccharide and intracellular polysaccharides. In infected macrophages, bioinformatics analyses revealed especially increased (hsa-miR-99b-3p) or decreased (hsa-miR-342-3p) miRNAs expression levels in response to infection with biofilms or both growth forms of *H. capsulatum* yeasts, respectively. The results of miRNAs suggested that infection by *H. capsulatum* can affect important biological pathways of the host cell, targeting two genes: one encoding a protein that is important in the cortical cytoskeleton; the other, a protein involved in the formation of stress granules. Expressed miRNAs in the host’s response could be proposed as new therapeutic and/or diagnostic tools for histoplasmosis.

## 1. Introduction

Histoplasmosis is an important systemic mycosis caused by the dimorphic ascomycete *Histoplasma capsulatum*. This pathogen can infect immunocompetent hosts exposed to infective propagules in highly contaminated places or cause an opportunistic infection in immunosuppressed individuals [1,2,3,4]. The highest occurrence of histoplasmosis is reported in hyper-endemic areas in North, Central, and South America, but it is also significant in Asia, Africa, and Australia [5].

Conidia and small hyphal fragments of the *H. capsulatum* mycelial phase constitute the major aerosolized infectious propagules found in the environment, which can be inhaled by susceptible hosts. Once in the host’s respiratory compartments are at 37 °C, the mycelial propagules convert to the parasitic yeast phase in approximately 1–3 h [6]. This facilitates an infection that can lead to a localized pulmonary disease or to a disseminated disease. Indeed, it is known that the transition from the mycelial phase to the parasitic yeast phase is required for *H. capsulatum* pathogenicity, and that the virulence of the fungal strains is associated with genes that are only expressed in the yeast phase [7,8,9,10,11].

The pathogenesis of histoplasmosis is relevant due to multiple virulence factors and the potential existence of fungal biofilm structures on medical devices or surgical implants, which could be associated with the detection of *H. capsulatum* infections in individuals with nosocomial risk factors, particularly in patients with endovascular histoplasmosis who have received vascular implants [12,13]. It is known that medical devices are subjected to the development of biofilms, which are multicellular communities held together by a self-produced extracellular matrix (ECM) [14]. Biofilm formation can facilitate pathogen infection, and those related to *H. capsulatum* infection have been recently investigated and partially characterized. The ability of this fungus to grow in biofilm form was firstly described by Pitangui et al. [15] by growing the yeast phase on an abiotic surface. Scanning electron microscopy revealed that *H. capsulatum* biofilm was a complex micro-organization with extracellular material linking several yeasts, resulting in a compact structure that persistently adhered to polystyrene plates. Furthermore, their particular metabolic activity was measured through the reduction of XTT tetrazolium salt (2,3-bis(2-methoxy-4-nitro-5-sulfophenyl)-5-[(phenylamino)carbonyl]-2H-tetrazolium-hydroxide) to XTT formazan [15], and proteomic analyses detected different protein patterns when comparing *H. capsulatum* in planktonic and biofilm conditions [16]. The susceptibility of the biofilm forms of *H. capsulatum* yeasts against an in vitro synergy of the N’-(1-phenylethylidene) isonicotinohydrazide compound and amphotericin B was evaluated by the checkerboard method, which revealed that mature biofilms were inhibited, approximately 50%, after treatment with the compound alone (100 × Minimal Inhibitory Concentration (MIC), 50× MIC, or 25× MIC, indistinctly). The combination formed by the compound (100× MIC) plus amphotericin B showed the best results, by allowing for the reduction of approximately 80% of mature biofilms [17]. Additionally, it was reported that mature biofilms were tested in vitro against antifungal agents such as itraconazole, amphotericin B, and farnesol. At lower concentrations (1.25× MIC), the antifungal drugs itraconazole and amphotericin B caused 20% and 15% inhibition, respectively, while farnesol caused the most pronounced inhibition (96%) of the biofilm metabolic activity [18].

There are many distinct molecules that can play regulatory roles in the host-pathogen interplay. Non-coding RNAs (ncRNAs) have been identified in several biological processes and diseases, mainly regulating gene expression [19,20,21,22,23]. Among the various types of ncRNAs, microRNAs (miRNAs) exhibit well-established regulatory functions by affecting mRNA stability [24], and their differential expression in host cells infected with *H. capsulatum* yeasts either in planktonic or biofilm growth forms could be used to understand the pathogenesis of histoplasmosis. MiRNAs are single-stranded RNA molecules of 19–23 nucleotides, generally originating in the cell nucleus. Once they reach the cytoplasm, they are targeted by complementary sequences in mRNAs transcripts, inducing cleavage or repressing post-transcriptional processing [25,26].

A few studies have explored the changes in miRNA expression in response to fungal infections. MiRNAs with significantly altered expression levels were reported in respiratory epithelial cells and murine macrophages infected with *Candida albicans* and in human monocytes and dendritic cells (DCs) infected with *Aspergillus fumigatus* [27,28,29,30]. Chen et al. [31] identified seven miRNAs in the THP-1 human monocyte cell line that were significantly upregulated in cells exposed for 6 h to *Cryptococcus neoformans* in comparison to cells at the starting point of the infection. In addition, De Lacorte Singulani et al. [32] identified eight miRNAs as novel biomarkers for paracoccidioidomycosis using reverse transcription-quantitative polymerase chain reaction (RT-qPCR). These circulating miRNAs, involved in the cellular processes of apoptosis and immune response, were differentially expressed in the serum of patients diagnosed with paracoccidioidomycosis.

Studies published by our research team have established a correlation between the infection mode of the *H. capsulatum* yeasts and biofilm formation [15,16,33]. Based on these previous findings, the aim of the present study was to detect differences in the miRNA expression between host cells infected with *H. capsulatum* yeasts from planktonic or biofilm cultures. Such findings could contribute to the development of future strategies in the elimination of this fungus from host tissues.

## 2. Materials and Methods

### 2.1. H. capsulatum Strains

The EH-315 strain, originally classified as a lone lineage by Kasuga et al. [34] and renamed by Teixeira et al. [35] as BAC1 phylogenetic species, was isolated from the intestine of an infected *Mormoops megalophylla* bat randomly captured in a cave in the state of Guerrero, Mexico. This bat species is not in danger of extinction; the procedures strictly complied with Mexican regulations on bat species protection, capture, and handling, and adhered to the ethical recommendations and guidelines of the American Society of Mammalogists published by Gannon and Sikes [36] for the use of wild mammals in research. This *H. capsulatum* strain was deposited in the *H. capsulatum* Culture Collection of the Fungal Immunology Laboratory, in the Department of Microbiology and Parasitology, at the School of Medicine of the National Autonomous University of Mexico (UNAM) (www.facmed.unam.mx/histoplas-mex/), and it was used solely for research purposes. This collection is registered in the World Federation for Culture Collections database under number LIH-UNAM WDCM817 (http://www.wfcc.info/ccinfo/index.php/strain/display/817/fungi/). The 60I strain was isolated from a mucocutaneous lesion in a human patient with a disseminated clinical form and it was deposited in the collection of the Clinical Mycology Laboratory of the Faculty of Pharmaceutical Sciences, UNESP, Brazil.

*H. capsulatum* yeasts were grown in a brain-heart infusion (BHI) broth (Difco Laboratories, Detroit, MI, USA) supplemented with 0.1% l-cysteine and 1% glucose at 37 °C for 24 h using rotary agitation (100 rpm).

Based on the determination of the lethal dose 50% (LD50) in six-week-old male inbred BALB/c mice, the EH-315 strain exhibited higher virulence (LD50 = 3 × 10^5^ cells/mL) than the 60I strain (LD50 = 3 × 10^8^ cells/mL) under controlled in vivo experimental conditions. These data were validated by an in vitro intracellular replication assay using the A549 pneumocyte cell line infected with the same *H. capsulatum* strains, where the number of viable intracellular yeasts was determined by a colony counting [Colony Forming Units (CFU)/mL ± standard deviation (SD)]. The results were: 5375 ± 1034, 11,667 ± 1527, and 64,200 ± 7116 for the EH-315 strain; and 3557± 1200, 7625 ± 2961, and 20,400 ± 600 CFU/mL for the 60I strain, considering 24, 48, and 72 h post-infection time points, respectively (ML Taylor, personal communication).

### 2.2. Ethics Statement

The experimental procedures with animals were approved by the Research and Ethics Committee of the Research Division at the UNAM. Protocol number-FM/DI/099/2017 was followed, in accordance with UNAM’s Animal Care and Use Committee recommendations and the Mexican Official Guide (NOM 062-ZOO-1999). Additionally, mice infection with *H. capsulatum* was approved by the School of Medicine Research and Ethics Committee (Project REF, PAPIIT-IN217418, 01/01/2018).

### 2.3. H. capsulatum Planktonic Cultures and Biofilm Formation

EH-315 and 60I yeasts were grown in a supplemented BHI broth in a 15 mL conical centrifuge tube (Falcon, BD Discovery Labware, Bedford, MA, USA) and incubated at 37 °C for 24 h with rotary agitation (100 rpm). Yeasts were harvested by low-speed centrifugation for 1 min at 600× *g* to remove large yeast clumps, suspended in 2 mL 0.01 M phosphate-buffered saline (PBS), pH 7.2, and a standard suspension of single yeasts was separated and adjusted to 5 × 10^6^ yeasts/mL, by counting in a Neubauer hemocytometer [33]. Afterwards, they were cultured in planktonic and biofilm growth forms.

For the planktonic growth form, yeasts were cultured in 250 mL Erlenmeyer flasks containing 100 mL fresh BHI broth, incubating them at 80 rpm in an orbital shaker at 37 °C, for 72 h. Finally, an infective inoculum was prepared using a yeast suspension adjusted to 5 × 10^6^ yeasts/mL in RPMI-1640 medium (Gibco, Life Technologies, Carlsbad, CA, USA).

For the biofilm formation, only the EH-315 yeasts were used. Biofilms were prepared according to Peeters et al. [37] with minor modifications by Pitangui et al. [15]. Initially, 500 μL of the single yeasts’ standard suspension (5 × 10^6^ yeasts/mL) was added to each well of a polystyrene 24-well plate (TPP, Trasadingen, Switzerland). The plate was incubated in an orbital shaker at 80 rpm, at 37 °C for 7 h for biofilm preadhesion. After, 2000 μL of the supplemented BHI broth were added to the wells, and the plate was incubated for 72 h for biofilm maturation. To prepare the infective inoculum from the biofilm growth form, yeasts were detached by means of surface scraping and harvested from each well of a single plate, washed in 0.01 M PBS, suspended in RPMI-1640 medium (Gibco), and adjusted to 5 × 10^6^ yeasts/mL.

### 2.4. Polysaccharide Extraction and Quantification

Production of soluble and insoluble exopolysaccharides (EPS and EPI) as well as intracellular polysaccharides (IP) was evaluated according to Da Silva et al. [36]. Briefly, *H. capsulatum* yeasts (5 × 10^6^ yeasts s/mL) were cultured in planktonic (EH-315 and 60I) and biofilm (EH-315) forms. Equivalent dry weight amounts of yeasts grown in planktonic form, mature biofilms, and supernatants of the biofilms were washed twice and transferred to plastic tubes containing PBS; each sample was then sonicated at 7 W for 30 s, centrifuged at 10,000× *g* for 5 min at 4 °C, and the supernatant containing the EPS fraction was collected. The pellet was treated with 1 M NaOH to extract the EPI fraction, which is vortexed for 15 min and centrifuged, after which the supernatant was collected. Finally, 1 M NaOH was added to the last residual pellet to extract IPs, the samples were heated at 100 °C for 15 min and centrifuged, and the supernatant was collected [38]. Three volumes of ice-cold ethanol were added to tubes containing EPS, EPI, and IP fractions. The tubes were frozen for 30 min at −20 °C and centrifuged, and the pellets were washed twice with cold 75% ethanol. Precipitated polysaccharides were suspended in 1 M NaOH [38]. The total carbohydrate amount was estimated by the phenol sulfuric method [39], using glucose as a standard. Color development was read spectrophotometrically at 490 nm.

The polysaccharide content of the EPS, EPI, and IP fractions was analyzed using the Prism 7.0 software (GraphPad Software Inc., La Jolla, CA, USA), and results were presented as mean values ± SD. Data were normalized by means of the Shapiro-Wilk test and analyzed using a one-way analysis of variance (ANOVA) followed by the Bonferroni’s multiple comparison test. Differences with *p* < 0.05 were considered statistically significant.

### 2.5. THP-1 Cell Culture and Differentiation

The THP-1 human monocyte cell line (ATCC TIB-202) was cultured in RPMI-1640 medium (Gibco), containing 10% fetal bovine serum (FBS), 5 mM glucose, 2 mM L-glutamine, 50 mg/mL gentamycin, and 20 mM HEPES (Sigma Chemical Co., St. Louis, MO, USA), at 37 °C and 5% CO_2_. To induce mature macrophages, herein called THP-1 Mø-like cells, a concentration of 5 × 10^5^ THP-1 cells/mL was treated with 30 ng/mL phorbol 12-myristate 13-acetate (PMA) (Sigma) for 24 h. Differentiated THP-1 Mø-like cells were washed with RPMI-1640 medium and incubated for 48 h in the same medium without PMA.

### 2.6. THP-1 Mø-Like Cell Viability Assay after H. capsulatum Yeast Infection

THP-1 Mø-like cells were infected with the EH-315 strain cultured in planktonic form and incubated at 37 °C for 5 h. The multiplicity of infection (MOIs) ratios were 10:1, 1:1, 1:10, and 1:100 yeasts:host cells, corresponding to concentrations of 5 × 10^6^, 5 × 10^5^, 5 × 10^4^, and 5 × 10^3^ yeasts/mL, respectively. To measure the viability of infected THP-1 Mø-like cells, the Live/Dead kit (Invitrogen, Thermo Fisher Scientific Inc., Waltham, MA, USA) was used following the manufacturer’s recommendations. Solutions of calcein AM and ethidium homodimer (EthD-1) were prepared and added to culture plates containing the infected THP-1 Mø-like cells. The plate was incubated for 30–45 min at room temperature and the images were acquired using the IN-Cell Analyzer 2000 apparatus (GE Healthcare Bio-Sciences Corp., Piscataway, NJ, USA). Cell viability was measured using the fluorescence of THP-1 Mø-like cells labeled with the Live/Dead kit and analyzed with the IN-Cell Investigator 1000 Workstation software (GE Healthcare Bio-Sciences Corp.). Statistical analyses were performed using the Prism 7.0 software. All assays were set up in triplicates, using duplicate samples per assay.

### 2.7. THP-1 Cells Infection

Differentiated THP-1 Mø-like cells were infected with a suspension of *H. capsulatum* yeasts, using an MOI of 10:1 with the EH-315 or 60I strains grown in planktonic cultures, and the EH-315 strain harvested from the biofilm culture. Uninfected cells were used as controls.

### 2.8. Determination of Infected THP-1 Mø-Like Cells Using Flow Cytometry

The number of THP-1 Mø-like cells infected with *H. capsulatum* was determined using the two different yeast growth forms of the EH-315 strain and the planktonic yeasts culture of the 60I strain. Yeasts were labeled with 5(6)-carboxy fluorescein diacetate N-succinimidyl ester (CFSE; Sigma); yeast suspensions were incubated with CFSE at 37 °C for 30 min. Monolayer cultures of THP-1 Mø-like cells were infected with an MOI of 10:1. The infected cells were incubated at 37 °C and 5% CO_2_ for 5 h. After incubation, the supernatant was removed and the infected cultures were trypsinized and resuspended in RPMI-1640 supplemented with FBS. Positive fluorescent-stained cells (with CFSE) were used to observe fungal cells that infected the THP-1 Mø-like cells. Size forward scatter (FSC), granularity side scatter (SSC), and fluorescence values were obtained by flow cytometry (FACSCanto^TM^, Becton & Dickinson, San Diego, CA, USA) with 10,000 THP-1 Mø-like cells per tube. The raw fluorescence intensity (FI) data of yeasts labeled with CFSE were analyzed using the FACSDiva^TM^ software version 6.1.3 (Becton & Dickinson). Uninfected THP-1 Mø-like cells, as well as labeled and unlabeled yeasts, were used as controls. The assay was set up in triplicates using duplicate samples per assay, and mean values from all the data were used for further analyses. Statistical analyses were conducted using the Prism 7.0 software and the results are presented as mean values ± SD. Data were normalized using the Shapiro-Wilk test and analyzed using ANOVA, followed by the Bonferroni’s multiple comparison test. Differences with *p* < 0.01 were considered statistically significant.

### 2.9. Total RNA Isolation, Quantification, and Integrity Assessment

The THP-1 Mø-like cells were lyzed with 2 mL of Trizol (Invitrogen, Carlsbad, CA, USA) and stored at −80 °C for RNA extraction. Total RNAs from infected and uninfected THP-1 Mø-like cells were extracted using the RNeasy Plant Mini kit (QIAGEN, Valencia, CA, USA), following the manufacturer’s instructions. RNA samples were quantified using a NanoDrop (Applied Biosystems, Thermo Fisher Scientific, Waltham, MA, USA) spectrophotometer.

The RNA extracted from THP-1 Mø-like cells interacting with different strains and growth forms of *H. capsulatum* yeasts and from uninfected cells was analyzed for integrity using the Agilent RNA 6000 Nano Kit on a 2100 Bioanalyzer (Agilent Technologies, Santa Clara, CA, USA) considering an RNA integrity number (RIN) of ≥ 8, according to the manufacturer’s recommendations.

### 2.10. qRT-PCR

For screening differentially expressed miRNAs in infected and uninfected THP-1 Mø-like cells, we implemented a qRT-PCR using human miRNome panels. The cDNA synthesis was performed using the Universal cDNA Synthesis kit II Exiqon (Exiqon A/S, Vedbaek, Denmark). The reaction was conducted in a thermocycler with one cycle of 60 min at 42 °C and 5 min at 95 °C. All generated cDNAs were stored at −20 °C until required.

Commercial 384-well plates were used for screening 752 human miRNAs available in human miRNome panels (microRNA Ready-to-Use PCR, Human panels I and II version 4, Exiqon A/S). Reaction mixtures containing cDNA, SYBR green qPCR Mastermix (Exiqon A/S), and RNase-free water were prepared and aliquoted in each well of the panels. Each sample was tested using panel I and panel II plates in triplicates, enabling the screening of the major known human miRNAs. The qRT-PCR analysis was performed using the ViiA™ 7 Real Time PCR System (Applied Biosystems), with one cycle of 10 min at 95 °C, followed by 45 amplification cycles of 10 s at 95 °C and 1 min at 60 °C for the determination of the melting curve. The expression levels of all miRNAs were calculated according to the ΔΔCq method [40], and the global normalization method was used to standardize the raw Cq values for each sample. Data were processed with the DataAssist version 3.01 software (Applied Biosystems) to determine miRNAs that were differentially expressed.

### 2.11. Bioinformatics and Statistical Analyses

All data generated from miRNA screening were analyzed using bioinformatics tools in order to identify functions and targets of the differentially expressed miRNAs. Comparative analyses were done using the five sets of pair comparisons, mentioned in the “differential microRNA expression” subsection of the Results section. Data analysis using the DataAssist version 3.01 software showed that the differences in miRNAs expression profiles between infected THP-1 Mø-like cells and uninfected controls were statistically significant, with *p* ˂ 0.05. DataAssist was also used for the construction of volcano plots for each of the comparative cell pairs in order to spot differences in miRNA expressions. A volcano plot is a scatter plot ionized to quickly identify changes in large data sets from replicate measurements; it plots significance on the y-axis versus fold change measured by relative quantification (RQ) on the x-axis.

For all differentially expressed miRNAs identified, in silico analyses allowed the recognition of cellular pathways in which they may participate and also the target genes that may be regulated by them. Pathway analyses were performed using the miRPath 2.0 software (http://diana.cslab.ece.ntua.gr/?sec=home) [41], which is a computational tool that identifies molecular pathways potentially altered by the expression of single or multiple miRNAs and the MIRSystem software (http://mirsystem.cgm.ntu.edu.tw/) [42], which simultaneously predicts target genes and their associated pathways for many miRNAs. In addition, analyses of the validated target genes regulated by the differentially expressed miRNAs were performed using miRWalk (http://www.umm.uni-heidelberg.de/apps/zmf/mirwalk/) [43], which is a complete database that provides information on human, mice, and rat miRNAs and validates target genes’ binding sites using the Kyoto Encyclopedia of Genes and Genomes (KEGG) database. Finally, association networks of miRNAs and their target genes were constructed using the Navigator software version 2.2 (Krembil Research Institute, Toronto, ON, Canada).

## 3. Results

### 3.1. Polysaccharide Matrix from H. capsulatum Yeasts

**Figure 1:**
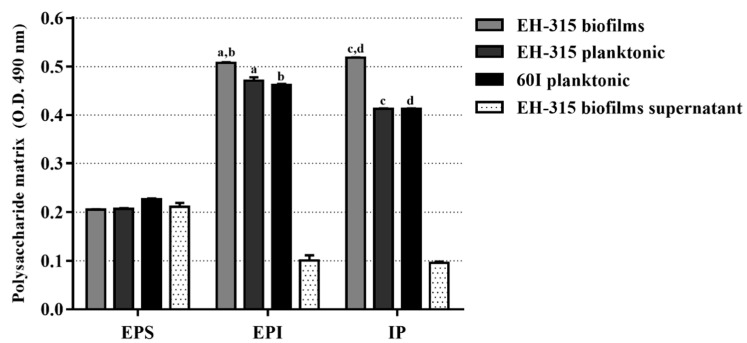
Polysaccharide matrix samples from *H. capsulatum* yeasts growing in planktonic culture and mature biofilm, and from the biofilm supernatant. a: *p* < 0.01, insoluble exopolysaccharides (EPI) produced by the EH-315 strain in planktonic culture vs. EPI produced by the EH-315 strain in biofilm form; b: *p* < 0.001, EPI produced by the 60I strain in planktonic culture vs. EPI produced by the EH-315 strain in biofilm form; c: *p* < 0.001, intracellular polysaccharides (IP) produced by the EH-315 strain in planktonic culture vs. IP produced by the EH-315 strain in biofilm form; d: *p* < 0.001, IP produced by the 60I strain in planktonic culture vs. IP produced by the EH-315 strain in biofilm form.

We quantified the total content of polysaccharide in samples from planktonic yeast cultures, mature biofilms, and biofilm supernatants. Results showed that the EPS content was similar among all samples, with no statistical differences. Concerning the EPI fraction, EH-315 yeasts grown in biofilm form had a statistically significant production (*p* < 0.0001), when compared to their planktonic cultures. The highest levels of IP were also produced by the EH-315 strain in biofilm form and were statistically significant (*p* < 0.0001) in relation to the IP produced by the EH-315 and 60I strains, under planktonic conditions (Figure 1). The quantification of polysaccharides in biofilm supernatants revealed the lowest levels of EPI and IP productions (Figure 1).

### 3.2. Viability of Infected THP-1 Mø-Like Cells

**Figure 2:**
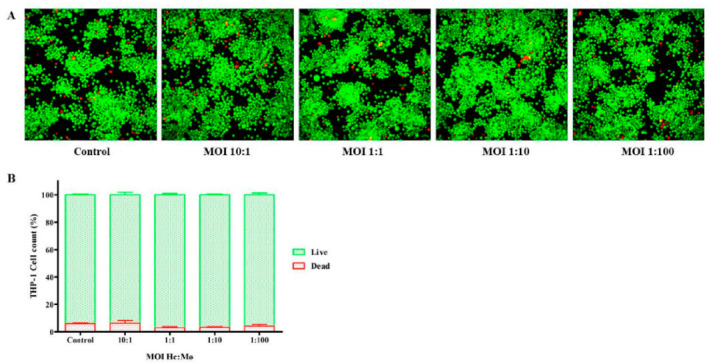
Viability of THP-1 Mø-like cells infected with *H. capsulatum* EH-315 yeasts 5 h after infection. (**A**) IN-Cell Analyzer images illustrating viable cells labeled by calcein AM (green) and dead cells labeled by EthD-1 (red). (**B**) Viability of the labeled cells obtained with the Live/Dead kit. Analyses were conducted with the IN-Cell Investigator 1000 Workstation software. Control = uninfected cells.

Figure 2 shows labeling data generated by the Live/Dead^®^ kit after the infection of THP-1 Mø-like cells with a suspension of *H. capsulatum* EH-315 yeasts at different MOIs. The images obtained with the IN-Cell Analyzer for all the MOIs, showed that most THP-1 Mø-like cells had an integral membrane and were stained in a green color, representing cell viability due to intracellular esterase activity (Figure 2A). The high viability of these cells was confirmed by fluorescence quantification: over 93.79% for all MOIs tested, with no statistical differences between the MOIs or between infected and uninfected controls (93.96%). Therefore, all of the employed MOIs were considered harmless as they produced THP-1 Mø-like cell viability values similar to those of the uninfected controls (Figure 2B).

### 3.3. Determination of Infected THP-1 Mø-Like Cells

**Figure 3:**
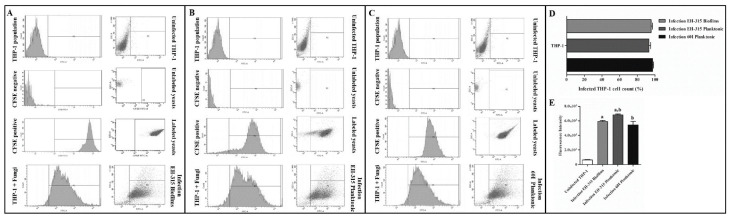
Analysis of THP-1 Mø-like cells-*H. capsulatum* yeasts interaction using flow cytometry. Flow cytometry profiles illustrating the fluorescence intensity (FI) of THP-1 Mø-like cells infected with (**A**) the EH-315 strain in biofilm cultures, (**B**) the EH-315 strain in planktonic cultures, and (**C**) the 60I strain in planktonic cultures 5 h after infection. Negative controls were uninfected cells (THP-1 cells population) and unlabeled yeasts (CFSE negative), while positive controls were fluorescently labeled yeasts (CFSE positive). THP-1 + Fungi represent the merged double population (THP-1 cells plus the fungal-stained CFSE). (**D**) Infection percentage and (**E**) FI (FITC-A mean) values demonstrate the interaction profile between THP-1 Mø-like cells and the EH-315 strain in biofilm and planktonic cultures or the 60I strain in planktonic cultures. The analyses were performed using a FACSCanto flow cytometer. The FI values were obtained from CFSE-labeled yeasts 5 h post-infection, by measuring 10,000 cells per tube, and they were analyzed using the FACSDiva software. Statistical data in graph E: (a) *p* < 0.01, THP-1 Mø-like cells infected with the EH-315 strain in planktonic cultures vs. THP-1 Mø-like cells infected with the EH-315 strain in biofilm cultures; (b) *p* < 0.0001, THP-1 Mø-like cells infected with the EH-315 strain in planktonic cultures vs. THP-1 Mø-like cells infected with the 60I strain in planktonic cultures.

The percentage of infected cells was estimated by analyzing the FI emitted by yeasts adhered to the THP-1 Mø-like cell membrane as well as by intracellular yeasts. Figure 3 shows the cytometric profiles generated by *H. capsulatum* yeasts from biofilm (Figure 3A, EH-315) and planktonic growth forms (Figure 3B, EH-315; Figure 3C, 60I) during the interaction with THP-1 Mø-like cells. Negative controls were unlabeled yeasts and uninfected cells, while positive controls contained fluorescently labeled cultured yeasts. According to the infection rate data (Figure 3D), the sampled *H. capsulatum* yeasts (EH-315 and 60I grown in planktonic cultures, as well as EH-315 grown in biofilms) yielded a significant percentage of infected THP-1 Mø-like cells 5 h after infection: 93.36 ± 1.89%, 95.74 ± 1.49%, and 97.38 ± 0.90%, respectively. In order to assess whether the different yeast strains and growth forms (planktonic or biofilm) of *H. capsulatum* might interfere with the FI of the infected cells, we implemented an additional analysis of the flow cytometry data. Results showed that the absolute FI mean values were different for the cells infected with the planktonic and biofilm yeast cultures from the EH-315 strain, and for those infected with the yeast planktonic culture from the 60I strain. Specifically, the FI mean values 5 h after infection were 6831 for cells infected with yeasts from the EH-315 strain grown in planktonic form and 5907 for cells infected with yeasts resulting from its biofilm, as well as 5434 for cells infected with yeasts from the 60I strain cultured in planktonic form (Figure 3E).

### 3.4. Quantification and Assessment of RNA Samples

Spectrophotometric analyses indicated that each RNA sample was obtained at a high concentration with a degree of purity consistent with standard values (A260/A280 1.8–2.0). The analysis of RNA integrity was satisfactory, presenting RINs greater than 8. Details on RNA concentrations, purity, and integrity are available in the supplementary material (Table S1; Figure S1).

### 3.5. Differential microRNA Expression

To generate as much information as possible and to compare the differences between overexpressed and repressed miRNAs, miRNAs expression profiles were analyzed between the following cell pairs: (1) THP-1 Mø-like cells infected with the EH-315 strain grown in planktonic form vs. uninfected THP-1 Mø-like cells; (2) THP-1 Mø-like cells infected with the 60I strain grown in planktonic form vs. uninfected THP-1 Mø-like cells; (3) THP-1 Mø-like cells infected with the EH-315 strain grown in biofilm form vs. uninfected THP-1 Mø-like cells; (4) THP-1 Mø-like cells infected with the EH-315 strain grown in biofilm form vs. THP-1 Mø-like cells infected with the EH-315 strain grown in planktonic form; and (5) THP-1 Mø-like cells infected with the EH-315 strain vs. THP-1 Mø-like cells infected with the 60I strain, both grown in planktonic form.

**Figure 4:**
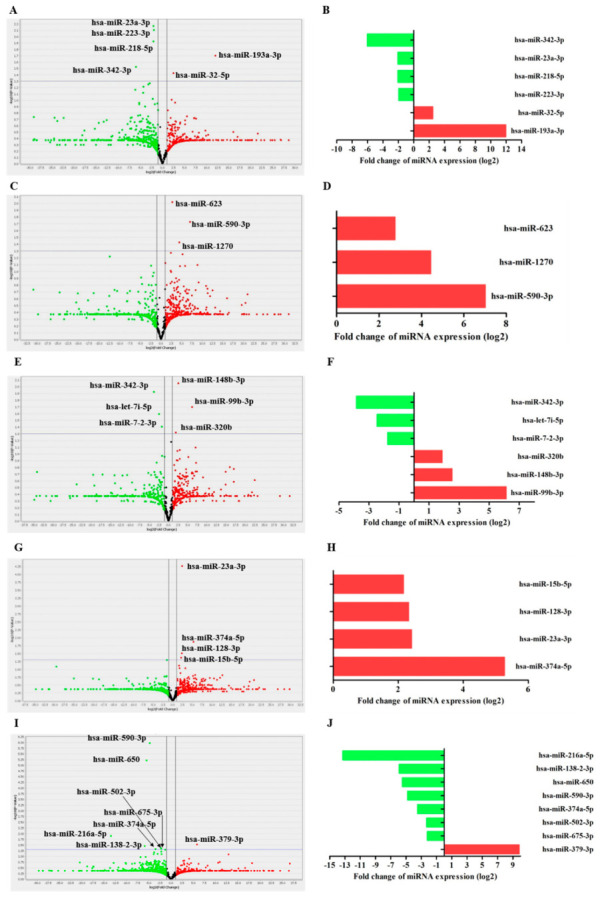
Volcano plot and fold change plot of miRNA regulation detected in THP-1 Mø-like cells infected with *H. capsulatum* yeasts. Differential analyses were performed between: (**A**,**B**) THP-1 Mø-like cells infected with the EH-315 strain grown in planktonic form vs. uninfected THP-1 Mø-like cells (control); (**C**,**D**) THP-1 Mø-like cells infected with the 60I strain grown in planktonic form vs. uninfected THP-1 Mø-like cells (control); (**E**,**F**) THP-1 Mø-like cells infected with the EH-315 strain grown in biofilm form vs. uninfected THP-1 Mø-like cells (control); (**G**,**H**) THP-1 Mø-like cells infected with the EH-315 strain grown in biofilm form vs. THP-1 Mø-like cells infected with the EH-315 strain grown in planktonic form; and (**I**,**J**) THP-1 Mø-like cells infected with the EH-315 strain vs. THP-1 Mø-like cells infected with the 60I strain, both grown in planktonic form. In the volcano plot, the x-axis shows the relative quantification (RQ) of each miRNA expression between each tested sample and its respective control in a log2 scale, while the y-axis shows the *p-*value for each miRNA adjusted in a log10 scale. Dots above the horizontal grey line indicate statistical significance (*p* ≤ 0.05). Spots in green indicate downregulated miRNAs and spots in red indicate upregulated miRNAs. The fold change plot shows RQ values of miRNAs expressed in the described samples and analyzed with the DataAssist version 3.01 software using the comparative method of Cq (ΔΔCq). Twenty-seven miRNAs exhibited significant differential expression (*p* ≤ 0.05).

Considering *p* = 0.05 as the cut-off value for significant differences, miRNAs with increased expression levels in the tested sample compared to the controls, indicated by RQ values ≥ 2 were considered upregulated, whereas miRNAs with decreased expression levels in relation to their controls, indicated by RQ ˂ 1, were considered downregulated. Figure 4 shows a volcano plot of differential analyses performed for each set of pair comparisons, identifying miRNAs with a significantly altered expression in the tested samples compared to their respective controls. Six miRNAs were differentially expressed in cells infected with EH-315 in planktonic form as compared to the uninfected controls, of which two (hsa-miR-32-5p and hsa-miR-193a-3p) were upregulated and four (hsa-miR-342-3p, hsa-miR-23a-3p, hsa-miR-218-5p, and hsa-miR-223-3p) were downregulated (*p* ≤ 0.05). Regarding the infection with the 60I strain in planktonic form, THP-1 Mø-like cells overexpressed three miRNAs (hsa-miR-623, hsa-miR-1270, and hsa-miR-590-3p) compared to the uninfected controls (*p* ≤ 0.05). Cells infected with EH-315 in biofilm form exhibited six differentially expressed miRNAs, of which three (hsa-miR-148b-3p, hsa-miR-99b-3p, and hsa-miR-320b) were upregulated and the other three (hsa-miR-342-3p, hsa-miR-7-2-3p, and hsa-let-7i-5p) were downregulated when compared to the uninfected controls (*p* ≤ 0.05).

Analysis of differential miRNAs expression profiles in THP-1 Mø-like cells infected with EH-315 in biofilm or planktonic forms revealed significant overexpression (*p* ≤ 0.05) of four miRNAs (hsa-miR-23a-3p, hsa-miR-374a-5p, hsa-miR-128-3p, and hsa-miR-15b-5p). In addition, from the comparison between miRNAs expression profiles of cells infected with planktonic forms of the EH-315 and 60I yeasts, eight miRNAs with statistically significant differential expressions (*p* ≤ 0.05) were identified, of which one (hsa-miR-379-3p) was overexpressed and seven (hsa-miR-590-3p, hsa-miR-650, hsa-miR-502-3p, hsa-miR-675-3p, hsa-miR-374a-5p, hsa-miR-216a-5p, and hsa-miR-138-2-3p) were repressed in THP-1 Mø-like cells infected with the EH-315 strain compared to the 60I strain. Table 1 shows all the differentially expressed miRNAs for each cell pair comparison, as well as their RQ values and biological functions. The levels of all differentially expressed miRNAs identified in the five sets of THP-1 Mø-like cell pairs analyzed were plotted as RQ in a log2 ratio scale and are shown in Figure 4.

### 3.6. MiRNA-mRNA Interactions

**Figure 5:**
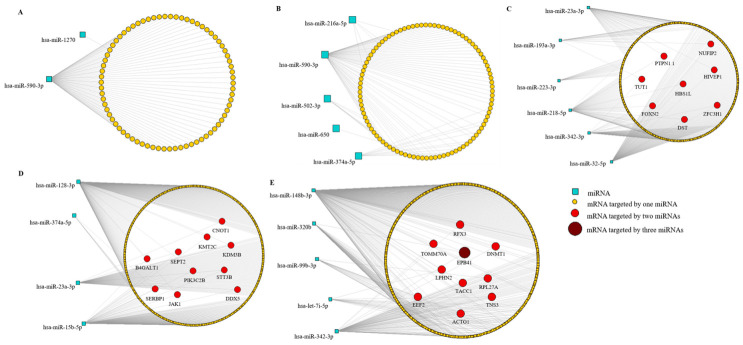
MiRNA-mRNA interaction networks to identify regulatory interactions between miRNAs and mRNA in cells infected with yeasts from distinct *H. capsulatum* strains. Interaction networks were constructed with the Navigator software v2.2 from triplicate analyses. Differentially expressed miRNAs were analyzed using the miRWalk software. MiRNAs are represented by squares and the target genes are represented through their mRNAs by circles. The grey lines connect miRNAs to their target mRNAs. Most mRNAs (yellow circles) are targeted by a single specific miRNA, whereas the remaining mRNAs, represented in red and dark red circles, are regulated by two or three distinct miRNAs, respectively. Analyses were performed between (**A**) THP-1 Mø-like cells infected with the 60I strain grown in planktonic form vs. uninfected THP-1 Mø-like cells (control); (**B**) THP-1 Mø-like cells infected with the EH-315 strain vs. THP-1 Mø-like cells infected with the 60I strain, both grown in planktonic form; (**C**) THP-1 Mø-like cells infected with the EH-315 strain grown in planktonic form vs. uninfected THP-1 Mø-like cells (control); (**D**) THP-1 Mø-like cells infected with the EH-315 strain grown in biofilm form vs. THP-1 Mø-like cells infected with the EH-315 strain grown in planktonic form; and (**E**) THP-1 Mø-like cells infected with the EH-315 strain grown in biofilm form vs. uninfected THP-1 Mø-like cells (control).

Differentially expressed miRNAs were integrated with their validated target genes using combined analyses from the miRWalk software in order to identify the regulatory interactions between miRNAs and mRNA. We constructed miRNA-mRNA networks and their interactions are displayed in Figure 5. Most mRNAs expressed from genes that were targeted by a single miRNA are shown in Figure 5A,B; Figure 5C,E show mRNAs from genes targeted by two distinct miRNAs; Figure 5E illustrates an mRNA potentially regulated by three miRNAs. In the differential analysis of THP-1 Mø-like cells infected with the EH-315 strain grown in planktonic form vs. uninfected THP-1 Mø-like cells, eight genes (*DST*, *FOXN2*, *HBS1L*, *HIVEP1*, *NUFIP2*, *PTPN1-1*, *TUT1*, and *ZFC3H1*) were potentially regulated at the same time by two distinct miRNAs (Figure 5C). When comparing THP-1 Mø-like cells infected with the EH-315 strain grown in biofilm form vs. uninfected cells, nine genes (*ACTG1*, *DNMT1*, *EEF2*, *LPHN2*, *RFX3*, *RPL27A*, *TACC1*, *TNS3*, and *TOMM70A*) were potentially downregulated by two miRNAs, while one gene (*EPB41*) was downregulated by three different miRNAs (Figure 5E). Regarding cells infected with the EH-315 strain grown in biofilm form in comparison to those infected with the EH-315 strain grown in planktonic form, 10 genes (*B4GALT1, CNOT1*, *DDX5*, *JAK1*, *KDM3B*, *KMT2C*, *PIK3C2B*, *SEPT2*, *SERBP1*, and *STT3B*) were potentially downregulated by two distinct miRNAs (Figure 5D).

### 3.7. In Silico Recognition of Signaling Pathways Hypothetically Regulated by Differentially Expressed miRNAs

The signaling pathways hypothetically changed in THP-1 Mø-like cells infected with yeasts from *H. capsulatum* as a result of differential miRNA expression are displayed in Table 2. According to our in silico analyses, in the cells infected with the EH-315 strain grown in planktonic form, the most relevant miRNA-affected pathways included those related to the fungus-host cell adhesion mechanisms, polysaccharide biosynthesis, regulation of cell structure and motility, protein degradation, response to proinflammatory stimuli, and regulation of cellular homeostasis. Regarding the cells infected with the 60I strain, overexpressed miRNAs could affect cell pathways associated with fungus-host cell adhesion mechanisms, polysaccharide biosynthesis, amino acid metabolism, and various processes regulating proliferation, apoptosis, cell differentiation, and migration. In the infection with the EH-315 strain derived from the biofilm growth form, numerous pathways would be changed in the infected cells (Table 2). Such pathways include fungus-host cell adhesion mechanisms, cell-cell interactions, polysaccharide biosynthesis, amino acid metabolism, processes regulating proliferation, apoptosis, cell differentiation and migration, responses to proinflammatory stimuli, and regulation of cellular homeostasis.

Finally, we compared miRNA expression in THP-1 Mø-like cells infected with different *H. capsulatum* strains (EH-315 vs. 60I) in planktonic cultures. The data indicated that differential miRNA expression in the cells infected with the EH-315 strain possibly regulates pathways related to fungus-host cell adhesion, proliferation, apoptosis, cell differentiation and migration, polysaccharide biosynthesis, lipid metabolism, amino acid metabolism, and protein degradation, as shown in Table 2.

### 3.8. In Silico Detection of Target Genes Regulated by Differentially Expressed miRNAs

**Figure 6:**
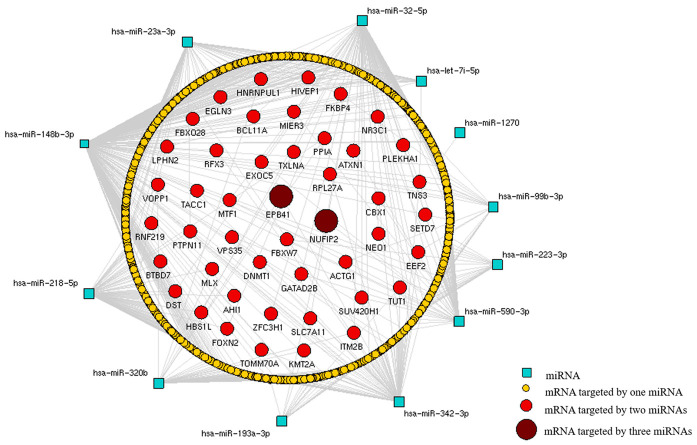
MiRNA-mRNA interaction networks to identify target genes regulated in cells infected with yeasts from distinct *H. capsulatum* strains. MiRNA-mRNA interaction networks identify target genes regulated in cells infected with different strains of *H. capsulatum*. The interaction network was constructed with the Navigator software v2.2 from triplicate analyses. Differentially expressed miRNAs were analyzed using the miRWalk software. Target genes regulated by differentially expressed miRNAs were analyzed in the following pair comparisons: THP-1 Mø-like cells infected with the EH-315 strain grown in planktonic form vs. uninfected THP-1 Mø-like cells, THP-1 Mø-like cells infected with the 60I strain grown in planktonic form vs. uninfected THP-1 Mø-like cells, and THP-1 Mø-like cells infected with the EH-315 strain grown in biofilm form vs. uninfected THP-1 cells. MiRNAs are represented by squares. Most genes indicated by their mRNAs (yellow circles) are targeted by a single specific miRNA, whereas the remaining genes (red and dark red circles) are simultaneously regulated by two or three distinct miRNAs.

To obtain target genes that were regulated in cells infected with yeasts from distinct *H. capsulatum* strains grown in different forms, an miRNA-mRNA interaction network was constructed. For this, we considered validated target genes tentatively regulated by the miRNAs with a significant differential expression in the following pairwise analyses: THP-1 Mø-like cells infected with yeasts from the EH-315 strain grown in planktonic form vs. uninfected cells; cells infected with yeasts from the 60I strain grown in planktonic form vs. uninfected cells; and cells infected with yeasts from the EH-315 strain grown in biofilm form vs. uninfected cells. These in silico analyses allowed the identification of common and relevant targets in the pathogenesis of histoplasmosis. The miRNA-mRNA network depicted in Figure 6 highlights two target genes (*EPB41* and *NUFIP2*) regulated by three distinct miRNAs among the analyzed samples. Notably, the miRNA-mRNA interaction network revealed that the expression of hsa-miR-148b-3p, hsa-miR-320b, and hsa-miR-342-3p miRNAs targeted the *EPB41* gene in the infected cells, whereas the expression of hsa-miR-32-5p, hsa-miR-590-3p, and hsa-miR-193a-3p miRNAs regulated the *NUFIP2* gene expression.

## 4. Discussion

MiRNAs have been proposed as stable biomarkers for various diseases, including mycoses [72]. Our results have particularly identified miRNAs involved in the regulation of host cell mechanisms triggered by the *H. capsulatum* infection, which could direct future strategies for the development of new therapeutic options and diagnostic tools for histoplasmosis.

Even though the EH-315 and 60I *H. capsulatum* strains were able to form biofilms, in accordance to Pitangui et al. [15], here, we selected the yeasts derived from the biofilm cultures of the EH-315 strain to test their ability to regulate miRNA expression, in infected THP-1 Mø-like cells. This selection was based on two distinctive characteristics of this strain, its highest virulence under in vivo and in vitro conditions, and its greatest likelihood to infect macrophages [33], both of which make this strain an optimal candidate for interacting with host cells.

Considering that EH-315 *H. capsulatum* yeasts grown in biofilms were evaluated by their interaction with macrophages, in the present study it was firstly important to confirm the biofilm structure of this strain. To do this, we quantified the polysaccharide matrix produced by *H. capsulatum* yeasts cultured in biofilm form, which reflects the amount of ECM present in the biofilm structure. ECM has been extensively studied due to its importance in maintaining the three-dimensional architecture of biofilms and its ability to act as a physical barrier protecting biofilm integrity and rendering it resistant to antifungal agents [73]. The results showed high amounts of EPI and IPs detected in the EH-315 yeasts grown in biofilm form, which are compatible with biofilm production.

In this study, we identified changes in the miRNAs expression profiles of THP-1 human macrophages infected with EH-315 or 60I *H. capsulatum* yeasts in planktonic form and EH-315 yeasts grown as biofilm. Based on volcano plot analyses, the THP-1 Mø-like cells infected with *H. capsulatum* strains in planktonic (EH-315 and 60I) and in biofilm form (EH-315) had different miRNAs expression profiles when compared to the controls of uninfected cells. Interestingly, our data suggest that even though the infection rates were similar in the EH-315 or 60I yeasts from planktonic cultures, each strain induced a particular gene regulation mechanism mediated by distinct miRNAs in the infected cells. Thus, we hypothesized that strain virulence could be directly related to the ability to induce changes in the expression of a specific miRNA. The present results highlighted the dramatic increases or decreases in host cell miRNAs when infected with different strains and growth forms of *H. capsulatum*.

A novel finding, not yet reported in systemic fungal infections, was associated with the hsa-miR-342-3p miRNA that was rapidly downregulated at 5 h after infection with either planktonic or biofilm cultures of the *H. capsulatum* EH-315 strain. Our analyses showed that hsa-miR-99b-3p was the only miRNA that was highly upregulated in cells infected with the EH-315 strain in biofilm form but not in planktonic form. Previous data published by Singh et al. [74] pointed to the role of miRNAs in *Mycobacterium tuberculosis* infection and pathogenesis, where miR-99b was substantially upregulated in infected DCs and macrophages. This strategy was adopted by *M. tuberculosis* to evade host immune responses, controlling TNF-α production and its survival within phagocytes. Additional assays confirmed that DCs treated with anti-miR-99b antagomirs showed a significant reduction in bacterial burden 48 h after infection when compared to the controls (74). Located in chromosome 19q13.41, the miR-99b gene produces the mature forms miR-99b-3p and miR-99b-5p, which are involved in several cellular activities such as cell proliferation, differentiation, and invasion [75]. Thus, we suggest that hsa-miRNA-99b-3p could be recommended as a new therapeutic target and as an important diagnostic tool for infections with *H. capsulatum* yeasts in biofilm form.

Usually, the regulatory effect of miRNAs on biological processes is attained by groups of these ncRNAs acting in a coordinated way. To understand the impact of the miRNAs disclosed in this study, it was necessary to identify cell pathways potentially affected by their differential expression. In fact, a great diversity of cellular pathways was tentatively regulated in THP-1 Mø-like cells after infection with *H. capsulatum* yeasts resulting from biofilms, thus highlighting the relevance of these yeast communities in the host intracellular microenvironment for histoplasmosis progression. Our data suggest that the Wnt and p53 signaling pathways might be affected by the differentially expressed miRNAs only in cells infected by *H. capsulatum* EH-315 grown as biofilms. Lina et al. [76] revealed the crucial role of the Wnt signaling pathway in the inhibition of lysosomal fusion and autolysosomal destruction in host cells infected with *Ehrlichia chaffeensis*, an obligate intracellular pathogen. Their findings demonstrate that *E. chaffeensis* exploits evolutionarily conserved Wnt signaling to inhibit autolysosome generation and autophagic destruction, promoting its intracellular survival. Here, we suggest that *H. capsulatum* yeasts organized in a biofilm structure induced the differential expression of miRNAs, affecting the Wnt signaling pathway as a strategy to evade the host immune defenses. Additionally, the p53 signaling pathway was also tentatively changed by the infection with *H. capsulatum* EH-315 yeasts in biofilm form. This pathway coordinates the cellular response to several types of stress, such as DNA damage and hypoxia, and its downstream signals allow for apoptosis, senescence, and cell cycle arrest [77,78]. Therefore, we suggest that the p53 pathway could be involved in infection progression since it could activate DNA repair proteins, induce cell cycle arrest, and initiate apoptosis if the infection caused irreparable cell damage.

Interestingly, infection of THP-1 Mø-like cells with the most virulent *H. capsulatum* strain (EH-315) affected the unsaturated fatty acids biosynthesis pathway. Lipid mediators are bioactive molecules produced in mammalian cells that can have anti-inflammatory roles, participate in inflammasome activation and cellular homeostasis regulation [79]. In the present study, our data suggest that the EH-315 strain induces a differential expression of miRNAs in THP-1 Mø-like cells that might affect fatty acid biosynthesis, possibly contributing to an alteration in the host immune response.

In general, the potentially affected pathways of THP-1 Mø-like cells in response to *H. capsulatum* yeast infection are related to fungus-host cell adhesion, as well as to the host’s inflammatory response and cell death, highlighting the ability of the yeasts to promote changes in the fundamental machineries of these phagocytic cells, and suggesting the involvement of fungal components or mechanisms in the pathogenesis of histoplasmosis.

The in silico analysis showed that the set of differentially expressed miRNAs identified in cells infected with the fungal strains grown in planktonic (EH-315 and 60I) or biofilm (EH-315) forms had two potential gene targets.

The first target gene, named *EPB41* (erythrocyte protein band 4.1), encodes the 4.1 protein, which is a component of the cortical cytoskeleton related to the cell membrane. Proteins of the 4.1 family are involved in the organization of cell polarity, as well as in cell adhesion and motility; they also play roles in transmembrane transport and in the response to growth factors. These proteins perform diverse functions because they connect components of the cortical cytoskeleton, such as actin, with transmembrane adhesion proteins, receptors, and transporters [80]. Here, we showed that infection with *H. capsulatum* yeasts induced the expression of the hsa-miR-148b-3p, hsa-miR-320b, and hsa-miR-342-3p miRNAs in the THP-1 Mø-like cells, efficiently and specifically regulating *EPB41*. This gene may contribute to the reorganization of the cytoskeleton, as well as to the regulation of yeast adhesion to the cell membrane and the components of the extracellular matrix, which are fundamental mechanisms in the pathogenesis of histoplasmosis.

The other target gene, *NUFIP2,* which encodes a nuclear fragile X mental retardation-interacting protein 2, is possibly regulated by the expression of hsa-miR-32-5p, hsa-miR-590-3p, and hsa-miR-193a-3p miRNAs through the infection of THP-1 Mø-like cells with *H. capsulatum* yeasts. According to Bish et al. [81], NUFIP2 is localized within stress granules in the cell cytosol after exposure to stress. Stress granules are dense ribonucleoprotein aggregates composed of translationally arrested mRNAs, ribosomal subunits, and proteins. These granules are generated as a result of different forms of cellular stress, including oxidative stress, heat shock, or nutrient deprivation, and they regulate mRNA stability and translation [82].

Overall, our data showed that THP-1 Mø-like cells infected with different *H. capsulatum* strains in different growth forms displayed specific miRNA signatures. These miRNAs are involved in the fate of the infected cells, since they can negatively regulate the expression of target genes at the mRNA level and interfere with several host cell pathways that participate in the pathogenesis of histoplasmosis.

We must underscore today’s prominence of technology that administers an interfering RNA (RNAi) to regulate or mimic the function of an miRNA in vivo, and which is based on the use of the so-called antagomirs or mimics. Antagomirs are characterized as single stranded antisense oligonucleotides used to inhibit miRNA’s function. This tool has demonstrated in vivo efficacy while acting on a miRNA and preventing a gene repression activity through the perfect complementarity between the oligonucleotide sequence administered and the target miRNA. Instead, therapeutic options using miRNAs are based on the synthetic delivery of a mimic miRNAs. Mimics are short double-stranded oligonucleotides, recognized and transported to the RNA-induced silencing complex in the cell cytoplasm, where the oligonucleotide can act as an endogenous miRNA by raising the level of the miRNA of interest and potentially blocking the expression of the target gene [83]. Studies using RNAi to identify virulence factors expressed by a particular target gene were improved in different microorganisms; of particular interest were those reports on *H. capsulatum* using RNAi to block genes associated with some virulence factors [84,85,86,87].

Finally, the participation of miRNAs generated by infected host cells in the control of host immune response was recently suggested as an immunotherapy tool for fungal infections [88].

## 5. Conclusions

In conclusion, we highlight the importance of a differentially expressed miRNA (hsa-miR-342-3p) in response to infection with both growth forms of *H. capsulatum* (planktonic and biofilm), and of another miRNA (hsa-miR-99b-3p) significantly overexpressed after infection with yeasts in biofilm form. These miRNAs could be recommended as new therapeutic and/or diagnostic tools in *H. capsulatum* infections displaying different clinical forms. The roles of these miRNAs in *H. capsulatum* infections of human patients should be further explored in order to better understand the pathogenesis of histoplasmosis.

## Figures and Tables

**Table 1:** Differentially expressed miRNAs in infected and uninfected THP-1 Mø-like cells. Relative quantification (RQ) values of miRNAs were determined with the DataAssist v3.01 software from triplicate measurements using the ΔΔCq method. RQ from downregulated miRNAs are shown in green and RQ from upregulated miRNAs are shown in red.

MiRNA ID	Fold Change (RQ)	*p* Value	Process or Function Categories Involved	References
**THP-1 Mø-like cells infected with the EH-315 strain vs. uninfected cells**				
hsa-miR-193a-3p	**3961.50**	0.0199	Inhibits cellular transformation	[44,45]
hsa-miR-32-5p	**5.50**	0.0373	Regulation of apoptosis	[46]
hsa-miR-223-3p	**0.25**	0.0078	Monocyte-macrophage differentiation; cell proliferation; Induction of apoptosis	[47,48]
hsa-miR-218-5p	**0.24**	0.0119	Inhibits cell cycle proliferation; induction of apoptosis	[49]
hsa-miR-23a-3p	**0.24**	0.0068	T-cell signaling; regulation of apoptosis	[50]
hsa-miR-342-3p	**0.01**	0.0297	Inflammation; pain signaling	[51]
**THP-1 Mø-like cells infected with the 60I strain vs. uninfected cells**				
hsa-miR-590-3p	**128.33**	0.01	Regulation of apoptosis	[52]
hsa-miR-1270	**21.65**	0.03	Regulation of IFNα1	[53]
hsa-miR-623	**6.69**	0.009	Cell proliferation, migration, and invasion of tumorous tissues	[54]
**THP-1 Mø-like cells infected with EH-315 strain biofilms vs. uninfected cells**				
hsa-miR-99b-3p	**69.97**	0.02	Cell proliferation	[55]
hsa-miR-148b-3p	**5.78**	0.008	Induce cell apoptosis and inhibiting cell invasion	[56]
hsa-miR-320b	**3.65**	0.04	Proliferation and cell invasion	[57]
hsa-miR-7-2-3p	**0.29**	0.03	Overexpression inhibits cell growth in lung cells line	[58]
hsa-let-7i-5p	**0.18**	0.02	Induces sensitivity to antineoplastic agents	[59]
hsa-miR-342-3p	**0.07**	0.01	Inflammation; pain signaling	[51]
**THP-1 Mø-like cells infected with EH-315 strain biofilms vs. cells infected with the EH-315 strain**				
hsa-miR-374a-5p	**38.54**	0.01	Hypoxia; Reduction of vascular permeability of lung tissue	[60,61]
hsa-miR-23a-3p	**5.32**	0.0001	T-cell signaling; regulation of apoptosis	[50]
hsa-miR-128-3p	**4.99**	0.03	Proliferation and cell invasion; apoptosis	[62]
hsa-miR-15b-5p	**4.46**	0.04	Downregulation of TNFα levels; apoptosis	[63]
**THP-1 Mø-like cells infected with the EH-315 strain vs. cells infected with the 60I strain**				
hsa-miR-379-3p	**903.56**	0.02	Regulation of cell adhesion; therapeutic response; drug resistance profiles	[64,65]
hsa-miR-675-3p	**0.21**	0.04	Migration and cell invasion	[66]
hsa-miR-502-3p	**0.20**	0.04	Inhibition of autophagy, cell growth and cell cycle progression	[67]
hsa-miR-374a-5p	**0.08**	0.04	Hypoxia; reduction of vascular permeability of lung tissue	[60,61]
hsa-miR-590-3p	**0.03**	0.0	Regulation of apoptosis	[52]
hsa-miR-650	**0.02**	0.0	Inhibits cell cycle progression; influences the proliferation capacity of B cells	[68]
hsa-miR-138-2-3p	**0.01**	0.03	Increases the proportion of early and late apoptosis; raises G1 phase arrest; and down-regulation of the S stage in the cell cycle	[69]
hsa-miR-216a-5p	**0.0001**	0.01	Decreases migration, invasion, cell viability and induces cell apoptosis	[70,71]

**Table 2:** Pathways associated with target genes regulated by differentially expressed miRNAs in Table 1. Mø-like cells infected with *H. capsulatum*. Data analyses used the miRPath 2.0 and MIRSystem software, along with the Kyoto Encyclopedia of Genes and Genomes (KEGG) database.

KEGG Pathway	Number of Genes	*P* Value
**THP-1 Mø-like cells infected with the EH-315 strain from planktonic growth form vs. uninfected cells**		
ECM-receptor interaction	14	1.1 × 10^−^^6^
Glycosaminoglycan biosynthesis	6	1.7 × 10^−^^12^
Ubiquitin mediated proteolysis	26	0.01
Focal adhesion	32	0.01
MAPK signaling (mitogen-activated protein kinase)	35	0.02
Apoptosis	12	0.04
Gap junction	17	0.04
Regulation of actin cytoskeleton	29	0.04
**THP-1 Mø-like cells infected with the 60I strain from planktonic growth form vs. uninfected cells**		
Glycosaminoglycan biosynthesis	7	1.9 × 10^−^^11^
ECM-receptor interaction	21	7.9 × 10^−^^11^
Lysine degradation	14	2.6 × 10^−^^5^
TGF-β signaling	21	0.0004
**THP-1 Mø-like cells infected with the EH-315 strain from biofilms growth form vs. uninfected cells**		
Glycosaminoglycan biosynthesis	7	2.1 × 10^−^^15^
ECM-receptor interaction	19	1.8 × 10^−^^10^
Lysine degradation	14	1.2 × 10^−^^6^
TGF-β signaling	26	2.1 × 10^−^^5^
Focal adhesion	49	0.001
Wnt signaling pathway	39	0.0002
Gap junction	19	0.009
Adherence junction	17	0.01
MAPK signaling	56	0.01
Apoptosis	18	0.01
N-Glycan biosynthesis	10	0.04
p53 signaling pathway	18	0.04
**THP-1 Mø-like cells infected with the EH-315 strain in biofilms growth form vs. cells infected with the EH-315 strain in planktonic growth form**		
Glycosaminoglycan biosynthesis	6	5.1 × 10^−^^41^
TGF-β signaling	26	3.4 × 10^−^^7^
p53 signaling pathway	22	0.0001
Wnt signaling pathway	38	0.0008
Focal adhesion	46	0.001
MAPK signaling	56	0.002
Ubiquitin mediated proteolysis	32	0.01
T cell receptor signaling pathway	25	0.02
**THP-1 Mø-like cells infected with the EH-315 strain vs. cells infected with the 60I strain, both in planktonic growth form**		
ECM-receptor interaction	26	2.6 × 10^−^^9^
TGF-β signaling	35	1.2 × 10^−^^8^
Glycosaminoglycan biosynthesis	7	1.5 × 10^−^^5^
Biosynthesis of unsaturated fatty acids	7	4.05 × 10^−^^5^
Lysine degradation	14	0.006
Ubiquitin mediated proteolysis	41	0.01
Valine, leucine, and isoleucine degradation	12	0.03

## Supplementary Materials

The following are available online at https://www.mdpi.com/2309-608X/7/1/60/s1, Figure S1: Electropherogram of RNA integrity. The analyses were performed by capillary electrophoresis on the Agilent 2100 Bioanalyzer^®^ system and the integrity of the RNA extracted from THP-1 Mø-like cells interacting with different strains and growth forms of *H. capsulatum* yeasts were evaluated and expressed as RIN values. RIN values of RNA extracted fromTHP-1 Mø-like cells infected with the *H. capsulatum* yeasts from EH-315 (**A**–**C**) and 60I (**D**–**F**) strains in planktonic growth cultures. RIN values of RNA extracted from THP-1 Mø-like cells infected with yeasts from EH-315 biofilms culture (**G**–**I**). RIN values of RNA extracted from uninfected THP-1 Mø-like cells used as control (**J**–**L**). All assays were performed in triplicate. Table S1: RNA concentration and purity. RNAs extracted from THP-1 Mø-like cells interacting with different strains and growth forms of *H. capsulatum* yeasts (planktonic cultures of the EH-315 and 60I strains, biofilms of the EH-315 strain), and uninfected THP-1 Mø-like cells (control), using the RNeasy Plant Mini Kit^®^ were quantified with a NanoDrop^®^. Data generated from single (*), duplicate (**), and triplicate (***) assays are shown.
