# Supplementary material for: Differential miRNA Expression in Human Macrophage-Like Cells Infected with Histoplasma capsulatum Yeasts Cultured in Planktonic and Biofilm Forms
Source: J Fungi (Basel). 2021 Jan 18;7(1):60. doi: 10.3390/jof7010060 (PMC7830537; doi:10.3390/jof7010060)
Supplement: Supplementary file 1 [file jof-07-00060-s001.zip › Response to Reviewer 1 Comments.docx]

**Response to Reviewer 1 Comments**

Comments and Suggestions for Authors

The authors of the manuscript entitled “Differential miRNA expression in human macrophage-like cells infected with Histoplasma capsulatum yeasts cultured in planktonic and biofilm forms” demonstrates differences in the mammalian miRNA expression between macrophages exposed to planktonic and biofilm yeasts. The authors identify several key miRNAs in the human macrophage-like line. The data with the miRNAs is new. However, there are multiple conceptual concerns regarding this manuscript.

1. The authors have previously written on several occasions about Histoplasma biofilms but as of yet they have never shown that this occurs in vivo. They and others have speculated that it might attach to plastic and cause infection on medically related devices but no such evidence exists. Histoplasma if it is a health care associated infection is most likely to spread by air handling systems (as was seen in UT Southwestern years ago) and not by the ability to lodge onto plastic. Thus, the effort made to study the differences between planktonic and biofilm has no basis in reality. If they can demonstrate that in tissues this organism does exist as a biofilm then that would be step forward. But until that time, there is no reason to even pursue the differences between planktonic and biofilm.

Answer to the Reviewer:

Thank you for your comments. In previous studies, we suggested that the biofilm formation *in vivo* by *H. capsulatum* yeasts could be related to this microorganism’s ability to avoid the host’s response, in contrast to what happens with single *H. capsulatum* yeasts, which are more easily eliminated by the host’s immune system than aggregated yeasts (Pitangui *et al.*, 2012; Pitangui *et al*., 2016). In a review, published by our team, a few advances were reported, which distinguished different patterns of proteins, when comparing *H. capsulatum* in planktonic and biofilm conditions. Using mass spectrometry, more than 40 proteins belonging to different functional groups were identified, as being differentially expressed either in biofilms or in dispersed yeasts (planktonic cultures), and the three main functional groups identified included proteins involved in the metabolism of amino acids, nuclear proteins, and translation proteins (Sardi *et al*., 2014). Our team is working on high-throughput methodologies that combine genomics, transcriptomics, proteomics, and metabolomics, to characterize the differential profile exhibited by fungi under planktonic and biofilm conditions. Advances in these methods should provide targets and/or biomarkers to identify the fungal biofilm in *H. capsulatum*-infected host cells. These approaches have been recently developed and are still at an analysis stage, and at present, we are working on the development of the preliminary steps, to better understand the pathogenic fungal biofilm associated to infections.

As you point out in your comment, undoubtedly, the infection caused by infective propagules spread in air handling systems is indeed the most frequent mechanism of *Histoplasma* infection; however, as we mentioned in our manuscript, contamination of medical devices in the hospital environment could also occur, even if in scarce situations. Moreover, there is interesting unpublished data, obtained by our team, showing that at 3, 24, and 48 hours post-infection, one can observe the presence of *H. capsulatum* yeast aggregates surrounding the macrophages’ nuclei in lung tissue sections of BALB/c mice intranasally infected with *H. capsulatum* micelial propagules of the LAm A/LAm A1 phylogenetic species (to be submitted). This finding matches those reported by Pitangui *et al*. (2016) for the alveolar murine macrophages AMJ2-C11 cell line.

References: Pitangui, N.S., Sardi, J.C., Silva, J.F., Benaducci, T., Moraes da Silva, R.A., Rodríguez-Arellanes, G., *et al*. (2012). Adhesion of *Histoplasma capsulatum* to pneumocytes and biofilm formation on an abiotic surface. Biofouling 28, 711-718. doi: 10.1080/08927014.2012.703659.

Sardi, J.C., Pitangui, N.S., Rodríguez-Arellanes, G., Taylor, M.L., Fusco-Almeida, A.M., and Mendes-Giannini, M.J.S. (2014). Highlights in pathogenic fungal biofilms. Rev Iberoam Micol. 31, 22–29. doi: 10.1016/j.riam.2013.09.014.

Pitangui, N. S. *et al*. An intracellular arrangement of *Histoplasma capsulatum* yeast-aggregates generates nuclear damage to the cultured murine alveolar macrophages. Front Microbiol 6, 1526 (2016).

2. The data with the miRNA for planktonic is fine but right now is merely descriptive. There is no effort to show that the changes in a miRNA really influence macrophage behavior. That needs to be done. There are certainly tools out there to inhibit or bolster expression of miRNAs so that work is not out of the realm of possibility. What is the impact of one of the miRNAs on macrophage behavior. It simply is not enough to describe the changes but rather it is important to describe what a particular miRNA may mean for handling of this fungus.

Answer to the Reviewer:

In answer to your question, it is important to highlight that all differentially expressed miRNAs were identified using a quantitative reverse transcription polymerase chain reaction (qRT-PCR) based Exiqon panel (human miRNome panel I, II, and V4; Exiqon A/S), in response to the infection with different *H. capsulatum* strains (EH-315 and 60I), under different growth form conditions (planktonic cultures and biofilms). This platform was also used by several authors in order to screen differentially expressed miRNAs in infected host macrophages (De Lacorte Singulani *et al*., 2017; Juracek *et al*., 2019; Nagy et al., 2019; Wu et al., 2018). Based on this “quantitative expressed data”, we identified 27 regulated miRNAs in host macrophages, after infection with *H. capsulatum* yeasts obtained from planktonic and biofilms culture conditions, as we mentioned in the Materials and Methods section. These results represent a miRNA signature infection, which may be a potential therapeutic target and/or diagnostic marker of histoplasmosis.

Our study is novel and original, because only few scientific publications have associated miRNA with fungal diseases (Croston *et al*., 2018; de Lacorte Singulani *et al*., 2020), and none with histoplasmosis. Moreover, for the first time, our study has shown the miRNA’s profile of infected host-cells in response to a *Histoplasma* challenge with strains differing in its virulence.

In regard to your question, “What is the impact of one of the miRNAs on macrophage behavior?”, as we mentioned in the Introduction section (Page 2, Lines 94-95), “Once they reach the cytoplasm, they are targeted to complementary sequences in mRNAs transcripts.” Additionally, we identified functions and validated target genes regulated by the differentially expressed miRNAs using an *in silico* analysis, in order to investigate the impact of expressed miRNAs in the behavior of infected host macrophages (according to the descriptions on Page 6, Lines 267-289, in the Materials and Methods section). Besides, in the Results section (Page 16, Lines 448-467), we reported the signaling pathways possibly changed in infected macrophages as a result of differential miRNAs expressions. Finally, we highlighted in the Discussion section (Page 21, Lines 542-546 and Page 22, Lines 547-574) that the infection of human macrophages with *H. capsulatum* yeasts promotes changes in the fundamental machineries of these phagocytic cells, particularly affecting pathways related to fungus-host cell adhesion, as well as to the host’s inflammatory response and cell death.

References: Croston TL, Lemons AR, Beezhold DH, Green BJ. (2018). MicroRNA Regulation of Host Immune Responses following Fungal Exposure. Front Immunol. 9:170. doi: 10.3389/fimmu.2018.00170.

De Lacorte Singulani J, De Fátima Da Silva J, Gullo FP, Costa MC, Fusco-Almeida AM, Enguita FJ, et al. (2017). Preliminary evaluation of circulating microRNAs as potential biomarkers in paracoccidioidomycosis. Biomed Rep. 6(3):353-357. doi: 10.3892/br.2017.849.

Singulani JL, Silva JFD, Gullo FP, et al. (2020) Fungal-host interactions: insights into microRNA in response to *Paracoccidioides* species. Mem Inst Oswaldo Cruz. 115:e200238. doi:10.1590/0074-02760200238

Juracek J, Piler P, Janku P, Radova L, Slaby O. (2019). Identification of microRNA signatures in umbilical cord blood associated with maternal characteristics. PeerJ. 7:e6981. doi: 10.7717/peerj.6981.

Nagy ZB, Barták BK, Kalmár A, Galamb O, Wichmann B, Dank M, et al. (2019). Comparison of Circulating miRNAs Expression Alterations in Matched Tissue and Plasma Samples During Colorectal Cancer Progression. Pathol Oncol Res. 25(1):97-105. doi: 10.1007/s12253-017-0308-1.

Wu J, Zhang H, Wang W, Zhu M, Qi LW, Wang T, et al. (2018). Plasma microRNA signature of patients with IgA nephropathy. Gene. 649:80-86. doi: 10.1016/j.gene.2018.01.050.

3. The use of a macrophage like line is ok to begin with. But it is vitally important that they extend these data to primary cells. They need to show that what they found with a cell line is true for primary cells. The cell line is derived from a single person with a single genetic composition. Whether what they find for a single cell line is applicable to primary cells from diverse individuals is unknown, but needs to be known.

Answer to the Reviewer: In response to your suggestion, we would like to clarify that in our countries (Brazil and Mexico), it is not possible to obtain primary cells from humans and, additionally, changing the study model to obtain primary cells from animals would not be compatible with the analyses performed. In our study, we investigated the miRNAs differential expressions in *H. capsulatum* infected human cells, hoping that miRNAs could be exploited as a diagnostic tool for histoplasmosis and, for this reason, we used human macrophages and a panel that allows the screening of human miRNAs.
