# Supplementary material for: Differential miRNA Expression in Human Macrophage-Like Cells Infected with Histoplasma capsulatum Yeasts Cultured in Planktonic and Biofilm Forms
Source: J Fungi (Basel). 2021 Jan 18;7(1):60. doi: 10.3390/jof7010060 (PMC7830537; doi:10.3390/jof7010060)
Supplement: Supplementary file 1 [file jof-07-00060-s001.zip › Response to Reviewer 2 Comments.docx]

**Response to Reviewer 2 Comments**

Comments and Suggestions for Authors

I found the article very well written and very detailed.

May i suggest in the discussion to perhaps expand on the implications for the group of patients with immunosuppression and disseminated histoplasmosis. how would these findings regarding miRNA expression be expected apply to a profoundly immunosuppressed HIV patient.

Answer to the Reviewer:

Thank you for your favorable comments about our manuscript. In response to your suggestion, to our knowledge, the expression of miRNAs in HIV patients has not been investigated until now. As far as we know, this is the first study to identify differentially expressed miRNAs in infected human cells with *H. capsulatum* yeasts. Recently, an investigation carried out by one of the co-authors of the present study (Taylor, ML) shows that analyses conducted on the bronchoalveolar lavage of immunosuppressed patients with HIV and histoplasmosis showed different cytokine profiles, but, so far, none of the investigations has aimed at finding an HIV-miRNAs correlation. We also searched for data in studies that have preliminarily characterized differentially expressed miRNAs, following exposure to clinically relevant fungal species; however, there is no data about the implications of miRNAs expressions for the group of immunosuppressed HIV patients (Monk *et al*., 2010; Agustinho *et al*., 2017; Chen *et al*., 2017; Croston *et al*., 2017; De Lacorte Singulani *et al*., 2017; Turini *et al*., 2017; Croston *et al*., 2018; Singulani *et al*., 2020). It was because of this lack of data, that the information recommended for inclusion was originally not considered in the Discussion section. In future, further studies by our research team could probably allow us to comply with your suggestion.

References

Agustinho DP, de Oliveira MA, Tavares AH, Derengowski L, Stolz V, Guilhelmelli F, et al. Dectin-1 is required for miR155 upregulation in murine macrophages in response to *Candida albicans*. Virulence (2017) 8(1):41–52. doi:10.1080/21505594.2016.1200215.

Chen H, Jin Y, Chen H, Liao N, Wang Y, Chen J. MicroRNA-mediated inflammatory responses induced by *Cryptococcus neoformans* are dependent on the NF-kappaB pathway in human monocytes. Int J Mol Med (2017) 39(6):1525–32. doi:10.3892/ijmm.2017.2951.

Croston TL, Lemons AR, Beezhold DH, Green BJ. (2018). MicroRNA Regulation of Host Immune Responses following Fungal Exposure. Front Immunol. 9:170. doi: 10.3389/fimmu.2018.00170.

Croston TL, Nayak AP, Lemons AR, Goldsmith WT, Germolec DM, Beezhold DH, et al. Pulmonary immune response following subchronic *Stachybotrys chartarum* exposure. J Allergy ClinImmunol (2017) 139(2):AB75. doi:10.1016/j.jaci.2016.12.291.

De Lacorte Singulani J, De Fátima Da Silva J, Gullo FP, Costa MC, FuscoAlmeida AM, Enguita FJ, et al. Preliminary evaluation of circulating microRNAs as potential biomarkers in paracoccidioidomycosis. Biomed Rep (2017) 6(3):353–7. doi:10.3892/br.2017.849 97.

Monk CE, Hutvagner G, Arthur JSC. Regulation of miRNA transcription in macrophages in response to *Candida albicans*. PLoS One (2010) 5(10):e13669. doi:10.1371/journal.pone.0013669.

Singulani JL, Silva JFD, Gullo FP, et al. (2020) Fungal-host interactions: insights into microRNA in response to *Paracoccidioides* species. Mem Inst Oswaldo Cruz. 115:e200238. doi:10.1590/0074-02760200238.

Turini Gonzales Marioto D, Navarro Dos Santos Ferraro AC, Goulart de Andrade F, Barros Oliveira M, Itano EN, Petrofeza S, et al. Study of differential expression of miRNAs in lung tissue of mice submitted to experimental infection by *Paracoccidioides brasiliensis*. Med Mycol (2017) 55:774–84. doi:10.1093/mmy/myw135 98.
