# Supplementary material for: Differential miRNA Expression in Human Macrophage-Like Cells Infected with Histoplasma capsulatum Yeasts Cultured in Planktonic and Biofilm Forms
Source: J Fungi (Basel). 2021 Jan 18;7(1):60. doi: 10.3390/jof7010060 (PMC7830537; doi:10.3390/jof7010060)
Supplement: Supplementary file 1 [file jof-07-00060-s001.zip › Response to Reviewer 3 Comments.docx]

**Response to Reviewer 3 Comments**

Comments and Suggestions for Authors

This is an impressively done paper containing both in vitro and in silico experiments. I have indicated some existing lacunae in the introduction, methods and presentation of results in the annotated PDF. I would recommend that the authors carefully revise the text of the manuscript for some occasional grammatical and punctuation errors. In addition, I would recommend another look at the discussion to improve the description of the in silico data and its biological significance. Overall, well done.

Answer to the Reviewer:

Thank you for your precise comments. And thank you for your suggestions, included in the PDF of the manuscript. We accepted most of them. Please, be advised that the changes have been included in the new original manuscript text. Below, we have specified where to find our answers to each specific question posed in the PDF format. Modifications in the manuscript can be seen in blue.

Page 2, Line 51: Please, see the modifications on Page 2, Line 51.

Page 2, Line, 55: Please, see the modifications on Page 2, Line 56.

Page 2, Line 60: Please, see the modifications on Page 2, Line 61.

Page 2, Lines 68-69: Please, see the modifications on Page 2, Line 70.

Page 2, Lines 75-78: Please, see the modifications on Page 2, Lines 76-86.

Page 2, Line 86: Please, see the modifications on Page 2, Lines 94 and 95.

Page 2, Line 88: Please, see the modifications on Page 2, Line 97.

Page 3, Line 105: Please, see the modifications on Page 3, Lines 116 and 117.

Page 3, Line 115: Please, see the modifications on Page 3, Line 128.

Page 3, Line 121: Please, see the modifications on Page 3, Lines 134-136.

Page 3, Line 129: The LD50 determination and the quantity of viable intracellular yeast are based on unpublished information obtained from a parallel study developed by some of our co-authors, which is part of a collaborative project between the UNESP’s and the UNAM’s research teams and, for which our co-authors are about to submit an article for publication. For this reason, we prefer not to describe the methodology used in this parallel study, but rather share these unpublished data as personal communication.

Page 3, Line 139: Please, see the modifications on Page 4, Line 156.

Page 4, Line 147: In response to your comment, we believe that it is important to highlight that in our current study, at the Multiplicity of Infection (MOI) assayed, high percentages of THP-1 Mø-like cells were infected by yeasts from planktonic cultures (EH-315 and 60I *H. capsulatum* strains) and yeasts derived from biofilm cultures (EH-315 strain). In this regard, although Pitangui *et al*. (2012) demonstrated that both strains of *H. capsulatum* (EH-315 and 60I) were able to form bioﬁlms, the EH-315 strain has unique characteristics that make it the best candidate to produce biofilms, interacting with THP-1 Mø-like cells, in order to reach further detection of miRNA expressions. These characteristics are related to its higher probability of infecting macrophages than that of the 60I strain, as referred by Pitangui *et al*. (2016); also, the EH-315 strain develops higher virulence, under *in vivo* and *in vitro* assays, than the 60I strain does. Please, see this information in the Discussion section, Page 21, Lines 501-506.

References:

Pitangui, N. S. *et al*. Adhesion of *Histoplasma capsulatum* to pneumocytes and biofilm formation on an abiotic surface. Biofouling 28, 711-8 (2012).

Pitangui, N. S. *et al*. An intracellular arrangement of *Histoplasma capsulatum* yeast-aggregates generates nuclear damage to the cultured murine alveolar macrophages. Front Microbiol 6, 1526 (2016).

Page 4, Line 153: Please, see the modifications on Page 4, Lines 168 and 169.

Page 4, Lines 162-165: Please, see the modifications on Page 4, Lines 181, 184 and 185.

Page 4, Line 168: Please, see the modifications on Page 4, Lines 185.

Page 4, Line 182: The heading of sub-section 2.5 of the Material and Methods was modified and a new sub-section 2.7 was included to describe the infection process of THP-1 Mϕ cells. Please, see the modifications on Page 4, Line 192 and on Page 5, Lines 215-218.

Page 5, Line 206: Please, see the modifications on Page 5, Lines 226 and 227.

Page 7, Line 278: In response to your precise suggestions, we explained notations a, b, c, and d in Figure 1 legend. Please, see the modifications on Page 7, Lines 301-308. Additionally, the Y axis title was also modified. Please, see the modifications in Figure 1. Moreover, concerning your questions about "dry weight OD," we prefer not to include any mention in Figure 1 legend, as we have addressed your questions in the Materials and Methods section. Please, see Page 4, Lines 171-191.

Page 8, Line 298: The acronym FI was described for the first time in the Materials and Methods section. Please, see Line 230 on Page 5.

Page 9, Line 322: As per your recommendations, letters a and b were maximized in panel E in Figure 3 (Page 9) and, in addition, notations a and b were described in Figure 3 legend (Page 9, Lines 350-352). Finally, you are correct in pointing out our omission of the quadrant markers in Panel A, which we inadvertently left out. Please, see the correction in Figure 3, panel A (Page 9).

Page 12, Line 383: In response to your question, we rechecked the zero P values in Table 1 and, as for the analysis performed by the DataAssist software, we can affirm that it is accurate. After revising Table 1, the authors noted an unintentional omission of one miRNA (has-miR-216a-5p), possibly due to a failure in the construction of this Table. We apologize for this omission. In the new version of the manuscript, this miRNA was included in Table 1.

Page 17, Line 430: Thank you for your comments. Please, note that according to Figure 4 and Table 1 (not Table 2), we identified eight miRNAs with significant differential expressions. Only one of them (hsa-miR-379-3p) was overexpressed, while seven of them (hsa-miR-216a-5p, hsa-miR-590-3p, hsa-miR-650, hsa-miR-502-3p, hsa-miR-675-3p, hsa-miR-374a-5p, and hsa-miR-138-2-3p) were repressed in THP-1 Mø-like cells infected with the EH-315 strain, as compared to infection by the 60I strain, in planktonic cultures. The bioinformatics analyses (Page 16, Lines 462-467) were related to pathway analyses performed with the miRPath 2.0 software and the MIRSystem software, which identify pathways potentially altered by **a** **set** of miRNAs (including eight miRNAs), irrespective of their overexpression or repression.

In attention to your suggestion we restructured the paragraphs that describe the *in silico* data in the Discussion section. Please, see the modifications on Page 22, Lines 577-596. In addition, we performed a careful English review in the manuscript text.
